# Functional impairment outcomes in clinical trials of different ADHD medications: post hoc responder analyses and baseline subgroup analyses

**DOI:** 10.1007/s00787-020-01586-5

**Published:** 2020-07-20

**Authors:** David R. Coghill, Tamara Werner-Kiechle, Sepehr Farahbakhshian, Caleb Bliss, Brigitte Robertson, Michael Huss

**Affiliations:** 1 Departments of Paediatrics and Psychiatry, Faculty of Medicine, Dentistry and Health Science, University of Melbourne, Melbourne, VIC 3010 Australia; 2 Murdoch Children’s Research Institute, Parkville, VIC Australia; 3 Shire, a Takeda company, Zug, Switzerland; 4 Shire, a Takeda company, Lexington, MA USA; 5 Child and Adolescent Psychiatry, Johannes Gutenberg-University Mainz, Mainz, Germany

**Keywords:** Attention-deficit/hyperactivity disorder, Weiss Functional Impairment Rating Scale-Parent, Functional impairment, Response

## Abstract

Several recent phase 3 clinical trials of attention-deficit/hyperactivity disorder (ADHD) medications have used the Weiss Functional Impairment Rating Scale-Parent Report (WFIRS-P). Here, we assess WFIRS-P response in individual patients in two pivotal trials of lisdexamfetamine dimesylate (LDX) and guanfacine extended release (GXR). We also analysed pooled WFIRS-P data from seven phase 3 studies of ADHD medications to shed light on factors associated with baseline functional impairment. The proportion of patients with a change in WFIRS-P score that exceeded the minimal important difference (MID) criteria for response was greater for LDX than placebo in the Family, Learning and School, and Risky Activities domains, and was greater for GXR than placebo in the Social Activities, Learning and School, and Family domains. Responders had significantly worse baseline scores in all WFIRS-P domains (all *p* < 0.001) than non-responders. In the pooled analyses, baseline WFIRS-P scores in all domains were significantly worse in participants with oppositional defiant disorder (ODD) than in those without ODD. Having combined type or hyperactive-impulsive type ADHD, being enrolled into a study in Europe, being male and being younger also had modest negative effects on baseline WFIRS-P scores. The present analysis of WFIRS-P response shows that previously reported group-level improvements in WFIRS-P functional impairment score translated into clinically relevant improvements in many individual participants. Functional impairment is a diverse and subjective construct that is influenced by multiple factors. Optimal management of individuals with ADHD should involve monitoring improvements in functioning and quality of life, as well as symptomatic improvement.

## Introduction

Attention-deficit/hyperactivity disorder (ADHD) is associated with functional impairment in multiple domains, such as daily life at home and at school [1, 2]. Therefore, as well as addressing the symptoms of ADHD, optimal treatment should also address the associated functional impairments [2]. To support this approach, the European Medicines Agency recommended that the endpoints of clinical trials of ADHD medications should reflect both symptomatic and functional outcomes [3].

The Weiss Functional Impairment Rating Scale-Parent Report (WFIRS-P) is a psychometrically validated parent-rated instrument for assessing functional impairment in children with ADHD [4–8], and has been used as an outcome measure in multiple phase 3 clinical trials of ADHD medications in children and adolescents [9–13]. The WFIRS-P assesses functional impairment typical of ADHD across six clinically relevant domains: Family; Learning and School (with subdomains of Learning and Behaviour); Life Skills; Child’s Self-Concept; Social Activities; and Risky Activities. Although these domains overlap with ADHD symptomatic impairment, functional impairment is a distinct construct [14]. As such, the WFIRS-P taps into distinct features not captured by symptom-based scales such as the ADHD Rating Scale IV (ADHD-RS-IV) [15]. Hodgkins et al. estimated the minimal important difference (MID) in WFIRS-P score for each domain [16]. The MID is a measure of the smallest change that a respondent would perceive as important and can be used to analyse the proportion of patients who respond to treatment.

The WFIRS-P was used in two pivotal randomized, double-blind, placebo-controlled, efficacy and safety studies of the ADHD treatments lisdexamfetamine dimesylate (LDX) and guanfacine extended release (GXR) [10]. Treatment with LDX was associated with statistically significant placebo-adjusted improvements from baseline to endpoint in WFIRS-P total score, and domain scores for Learning and School, Family, Social Activities, and Risky Activities (all *p* < 0.001). In the same study, treatment with the active reference arm osmotic-release oral system methylphenidate (OROS-MPH) showed a similar pattern of improvement [10]. In the GXR study, placebo-adjusted improvements from baseline to endpoint were statistically significant in the WFIRS-P Learning and School, and Family domains (both *p* < 0.01) [11]. Similar improvements were seen with the atomoxetine (ATX) reference arm [11].

The present analyses were conducted to enhance understanding of the clinical relevance of previously reported group-level WFIRS-P outcomes from clinical trials of ADHD medications. First, using the anchor-based WFIRS-P MID calculated by Hodgkins et al. [16], we assessed individual treatment response in two pivotal trials of LDX and GXR which included OROS-MPH and ATX, respectively, as reference arms [10, 11]. Then, using pooled WFIRS-P data from seven phase 3 studies of ADHD medications, we explore demographic and clinical factors which may impact functional impairment in clinical trial participants at baseline.

## Methods

### WFIRS-P treatment response analysis

#### Study designs and populations

Studies SPD489-325 and SPD503-316 were chosen for post hoc analysis of individual response because both have previously published group-level effect sizes in WFIRS-P domain and total scores for active and reference arms [10, 11].

SPD489-325 (ClinicalTrials.gov identifier: NCT00763971) was a 7-week, double-blind, efficacy and safety study of LDX in children and adolescents (aged 6–17 years) with ADHD and an ADHD-RS-IV total score of at least 28, conducted in Europe (48 sites in 10 countries). Details of the study design, results and previous post hoc analyses have been published [10, 15, 17–21].

SPD503-316 (ClinicalTrials.gov identifier: NCT01244490) was a 10–13-week, double-blind, efficacy and safety study of GXR in children and adolescents (aged 6–17 years) with ADHD and an ADHD-RS-IV total score of at least 32 and a Clinical Global Impression-Severity (CGI-S) score of at least four, conducted in Europe (45 sites in 11 countries) and North America. Details of the study design, results and previous post hoc analyses have been published [11, 15, 22].

Both studies were placebo-controlled and included an active reference arm. In SPD489-325, participants were randomized (1:1:1) to receive dose-optimized LDX (30, 50 or 70 mg/day), placebo or OROS-MPH (18, 36 or 54 mg/day). In SPD503-316, participants were randomized (1:1:1) to receive dose-optimized GXR (children, 1–4 mg/day; adolescents, 1–7 mg/day), placebo or ATX (10–100 mg/day). The studies were neither designed nor powered for comparisons between active treatments.

#### Pre-specified WFIRS-P analyses

Summaries of the pre-specified efficacy analyses from SPD489-325 and SPD503-316 are provided for context. In both studies, efficacy analyses were carried out using the full analysis set (FAS), defined as all participants who received at least one dose of investigational product. Least-squares means, effect sizes, and *p* values were based on type III sum of squares from an analysis of covariance model for the change from baseline, including treatment group, age group, and country as a fixed effect, and baseline value as covariates [10, 11]. Except for the pre-specified key secondary outcomes in SPD503-316 (the Family domain, and the Learning and School domain), *p* values were not adjusted for multiplicity and are therefore non-inferential.

#### Post hoc WFIRS-P responder analyses

WFIRS-P response in each of the six domains was defined as a change exceeding the published MID for that domain (Family, 3.76; Learning and School, 3.94; Life Skills, 3.59; Child’s Self-Concept, 1.28; Social Activities, 2.78; Risky Activities, 2.60) [16]. *p* values were not adjusted for multiplicity.

To investigate the impact of the degree of impairment at baseline on the magnitude of improvement during the study, post hoc analyses were undertaken to compare baseline WFIRS-P scores in responders and non-responders for each domain, and to assess correlation between baseline WFIRS-P score and score change from baseline in that domain. Participants in the FAS with data at both baseline and endpoint were assessed for studies SPD489-325 and SPD503-316. The FAS was chosen as the analysis population for consistency with previously reported studies, in which it was used as the primary population for efficacy analyses.

### Factors associated with WFIRS-P scores at baseline in seven clinical trials

#### Study designs

To explore potential factors that might be negatively associated with WFIRS-P scores at baseline (before treatment), we pooled data from all LDX and GXR studies that included a baseline assessment using the WFIRS-P.

As well as the two studies described above (SPD489-325 and SPD503-316), LDX studies SPD489-326 and SPD489-317, and GXR studies SPD503-315, SPD503-314 and SPD503-312 were included. All were randomized, double-blind, placebo-controlled studies, except for SPD489-317 which was a randomized, double-blind, head-to-head study with ATX. Key study information, and inclusion and exclusion criteria are summarized in Table 1.

**Table 1:** Key inclusion and exclusion criteria for studies in the pooled WFIRS-P data set

Study number (Clinicaltrials.gov identifier)	Drug	Study design	Location	Reference arm	ADHD-RS-IV total score	CGI-I score	Notable inclusion/exclusion criteria
SPD489-325 [17] (NCT00763971)	LDX	7-week, double-blind study in children and adolescents (aged 6–17 years)	Europe	OROS-MPH	≥ 28	NA	Individuals who had not responded previously to OROS-MPH were excluded Enrolment was managed to ensure that ≥ 25% of participants were adolescents (aged 13–17 years)
SPD489-326 [30] (NCT00784654)	LDX	Randomized withdrawal study in children and adolescents (aged 6–17 years); a 26-week open-label period followed by a 6-week double-blind withdrawal period	Europe/North America	None	≥ 28	NA	Individuals who had not responded previously to OROS-MPH were excluded
SPD489-317 [31] (NCT01106430)	LDX	9-week, double-blind, head-to-head efficacy study in children and adolescents (aged 6–17 years)	Europe/North America	ATX	≥ 28	NA	All participants had demonstrated a previous inadequate response to MPH Individuals were excluded if they had previously been exposed to ATX or AMF, had experienced intolerable side effects with MPH, had failed to respond to > 1 course of MPH or had been treated with > 1 formulation of MPH
SPD503-315 [32] (NCT01081145)	GXR	Randomized withdrawal study in children and adolescents (aged 6–17 years); a 13-week, open-label dose-optimization period followed by a 26-week double-blind withdrawal period	Europe/North America	None	≥ 32	≥ 4	
SPD503-316 [11] (NCT01244490)	GXR	7-week, double-blind study in children and adolescents (aged 6–17 years)	Europe/North America	ATX	≥ 32	≥ 4	Enrolment was managed to ensure that ≥ 25% of participants were adolescents (aged 13-17 years) and ≥ 25% of participants were girls
SPD503-314 [33] (NCT00997984)	GXR	8-week, double-blind efficacy study of GXR, taken by children (aged 6–12 years) in either the morning or evening	North America	None	≥ 28	≥ 4	Enrolment was managed to ensure that ≥ 25% of randomized participants were girls
SPD503-312 [13] (NCT01081132)	GXR	13-week, double-blind study of GXR in adolescents (aged 13–17 years)	North America	None	≥ 32	≥ 4	

In all studies, participants were excluded if symptoms were well controlled with acceptable tolerability on their current ADHD medication, or if they had a comorbid psychiatric diagnosis except for oppositional defiant disorder

*ADHD* attention-deficit/hyperactivity disorder, *ADHD*-*RS*-*IV* Attention-Deficit/Hyperactivity Disorder Rating Scale IV, *AMF* amphetamine, *ATX* atomoxetine, *CGI*-*I* Clinical Global Impression-Improvement, *GXR* guanfacine extended release, *LDX* lisdexamfetamine dimesylate, *MPH* methylphenidate, *NA* not applicable, *OROS*-*MPH* osmotic-release oral system methylphenidate, *WFIRS*-*P* Weiss Functional Impairment Rating Scale-Parent Report

#### Post hoc pooled baseline stratifications and correlations

The FAS from each study was pooled. Defined as all participants who received at least one dose of investigational product, the FAS was chosen as the analysis population for consistency with previously reported studies.

Participants were stratified into subgroups based on: presence of oppositional defiant disorder (ODD, with or without); presence or absence of hyperactivity (‘with hyperactivity’ included predominantly hyperactive-impulsive and combined type ADHD, ‘without hyperactivity’ included predominantly inattentive type ADHD); continent of enrolment (Europe or North America); and sex (male or female). Standardized mean differences (SMD) between subgroups were calculated as Hedges *g* (SMD ≥ 0.8 was considered large; SMD ≥ 0.5 was considered medium; SMD ≥ 0.2 was considered small) [23]. The effect of participants’ ages was investigated using linear regression (Pearson’s *r*). Only *p* values below 0.001 are quoted. Subgroup categories were selected based on a standard set of baseline clinical and demographic factors that were pre-defined in all seven studies.

## Results

### WFIRS-P treatment response

#### Participant disposition and characteristics

In the SPD489-325 study, 336 participants were randomized and 317 were included in the FAS, of whom 80/104, 42/106 and 74/107 in the LDX, placebo and OROS-MPH groups, respectively, completed the study [17]. The mean age of participants in the FAS was 10.9 years (standard deviation [SD], 2.70); 72.2% were children aged 6–12 years and 80.4% were boys. Overall, 15.8% of participants had predominantly inattentive ADHD, 3.2% had predominantly hyperactive-impulsive ADHD and 81.0% had combined type ADHD. ODD affected 7.7% of the LDX group, 7.5% of the placebo group and 9.3% of the OROS-MPH group. Stimulant medication was previously used by 47.1%, 45.3% and 48.6% of the LDX, placebo and OROS-MPH groups, respectively (Table 2).

**Table 2:** Baseline demographics and disease characteristics (FAS)

Characteristic	SPD489-325			SPD503-316		
	LDX (*n* = 104)	Placebo (*n* = 106)	OROS-MPH (*n* = 107)	GXR (*n* = 114)	Placebo (*n *= 111)	ATX (*n* = 112)
Age, years						
Mean (SD)	10.8 (2.78)	11.0 (2.77)	10.7 (2.56)	10.9 (2.77)	11.0 (2.76)	10.5 (2.81)
Median (range)	11.0 (6–16)	11.0 (6–17)	11.0 (6–16)	11.0 (6–17)	11.0 (6–17)	10.0 (6–16)
Sex [*n* (%)]						
Male	81 (77.9)	88 (83.0)	86 (80.4)	76 (66.7)	86 (77.5)	87 (77.7)
Continent of enrolment [*n* (%)]						
Europe	104 (100)	106 (100)	107 (100)	88 (76.5)	87 (78.4)	87 (77.7)
ADHD type [*n* (%)]						
Predominantly inattentive	22 (21.2)	15 (14.2)	13 (12.3)	15 (13.2)	11 (9.9)	10 (8.9)
Predominantly hyperactive-impulsive	2 (1.9)	7 (6.6)	1 (0.9)	6 (5.3)	5 (4.5)	3 (2.7)
Combined	80 (76.9)	84 (79.2)	92 (86.8)	93 (81.6)	95 (85.6)	99 (88.4)
ODD diagnosis [*n* (%)]	8 (7.7)	8 (7.5)	10 (9.3)	17 (14.9)	14 (12.6)	10 (8.9)
Previous stimulant medication use	49 (47.1)	48 (45.3)	52 (48.6)	54 (47.4)	56 (50.5)	57 (50.9)

*ADHD* attention-deficit/hyperactivity disorder, *ATX* atomoxetine, *FAS* full analysis set, *GXR* guanfacine extended release, *LDX* lisdexamfetamine dimesylate, *ODD* oppositional defiant disorder, *OROS*-*MPH* osmotic-release oral system methylphenidate, *SD* standard deviation

In the SPD503-316 study, 338 participants were randomized and 337 were included in the FAS, of whom 91/114, 92/111 and 89/112 in the GXR, placebo and ATX groups, respectively, completed the double-blind period of the study [11]. The mean age of participants in the FAS was 10.8 years (SD, 2.78), 71.8% were children aged 6–12 years and 73.9% were boys. Overall, 10.7% had predominantly inattentive, 4.2% had predominantly hyperactive-impulsive and 85.2% had combined type ADHD. In total, 12.2% had comorbid ODD and 56.3% had significant oppositional symptoms. Stimulant medication was previously used by 47.4%, 50.5% and 50.9% of the GXR, placebo and ATX groups, respectively (Table 2).

In both studies, the principal reason for discontinuation was lack of efficacy. Baseline demographics and disease characteristics were generally similar across treatment groups (Table 2).

#### WFIRS-P outcomes and responder analyses in SPD489-325

**Fig. 1:**
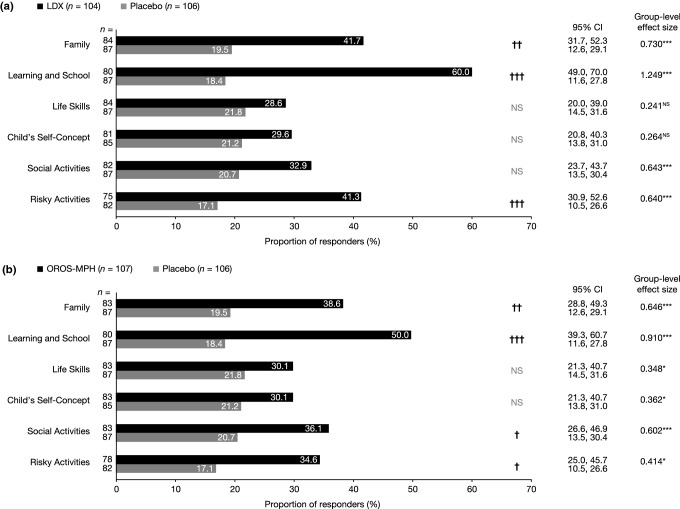
SPD489-325: post hoc responder analysis of change in WFIRS-P domain scores in the **a** LDX and placebo groups and **b** OROS-MPH and placebo groups. *p < 0.05; ***p < 0.001; analysis of covariance (pre-specified) [10]. ^†^*p *< 0.05; ^††^*p *< 0.01; ^†††^*p *< 0.001; χ^2^ test (post hoc). All *p* values are nominal and not adjusted for multiplicity. Numbers of observations (*n*) for each group are shown for each WFIRS-P domain. WFIRS-P response was defined as a change from baseline to endpoint exceeding the published MID [16]. *CI* confidence interval, *LDX* lisdexamfetamine dimesylate, *MID* minimum important difference, *NS* not significant, *OROS*-*MPH* osmotic-release oral system methylphenidate, *WFIRS*-*P* Weiss Functional Impairment Rating Scale-Parent Report

The proportion of patients with a change in WFIRS-P score that exceeded the MID was significantly greater for LDX than placebo in the Family, Learning and School, and Risky Activities domains (Fig. 1a). Mean improvements in WFIRS-P scores from baseline to endpoint were significantly greater for LDX than for placebo in the Learning and School, Family, Social Activities, and Risky Activities domains, with effect sizes of 1.249, 0.730, 0.643 and 0.640, respectively (Fig. 1a), as previously described [10].

In the OROS-MPH reference arm, the proportion of participants with a change in WFIRS-P score exceeding the MID was significantly greater than for placebo in the Family, Learning and School, Social Activities, and Risky Activities domains (Fig. 1b). Placebo-adjusted improvements in mean WFIRS-P scores were significant for OROS-MPH in all domains, with effect sizes in the range 0.348–0.910 (Fig. 1b), as previously described [10].

#### WFIRS-P outcomes and responder analyses in SPD503-316

**Fig. 2:**
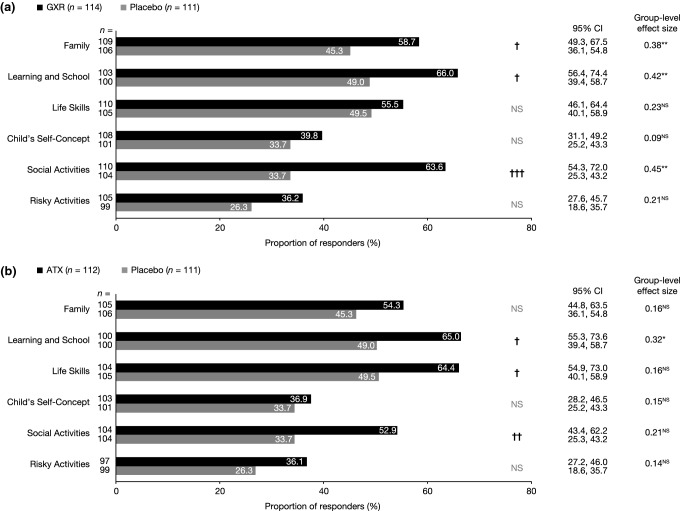
SPD503-316: post hoc responder analysis of change in WFIRS-P domain scores in the **a** GXR and placebo groups and **b** ATX and placebo groups. *p < 0.05; **p < 0.01; analysis of covariance (pre-specified). ^†^*p *< 0.05; ^††^*p *< 0.01; ^†††^*p *< 0.001; *χ*^2^ test (post hoc). All *p* values are nominal and not adjusted for multiplicity, except for the pre-specified analysis of the Family domain and the Learning and School domain. Numbers of observations (*n*) for each group are shown for each WFIRS-P domain. WFIRS-P response was defined as a change from baseline to endpoint exceeding the published MID [16]. *ATX* atomoxetine, *CI* confidence interval, *GXR* guanfacine extended release, *MID* minimum important difference, *NS* not significant, *WFIRS*-*P* Weiss Functional Impairment Rating Scale-Parent Report

The proportion of patients with a change in WFIRS-P score that exceeded the MID was greater for GXR than placebo in the Social Activities, Learning and School, and Family domains (Fig. 2a). Placebo-adjusted improvements in mean WFIRS-P scores from baseline to endpoint were significant for GXR in the Social Activities, Learning and School, and Family domains, with effect sizes of 0.45, 0.42 and 0.38, respectively (Fig. 2a), as previously described [11].

In the ATX reference arm, the proportion of participants with a change in WFIRS-P score exceeding the MID was significantly greater than for placebo in the Social Activities, Learning and School, and Life Skills domains (Fig. 2b). Placebo-adjusted improvements in mean WFIRS-P scores were significant in the Learning and School domain only, with an effect size of 0.32 (Fig. 2b), as previously described [11].

#### Post hoc analysis of WFIRS-P response

In both studies, responders had significantly worse baseline scores in all WFIRS-P domains (all *p* < 0.001) than non-responders (Table 3). In all domains, baseline WFIRS-P scores were significantly correlated with the change in scores from baseline (all *p* < 0.001), with greater baseline impairment associated with larger score changes.

**Table 3:** Summary of baseline WFIRS-P scores for responders and non-responders in studies SPD489-325 and SPD503-316

WFIRS-P domain	SPD489-325 baseline WFIRS-P score, mean (SD)		SPD503-316 baseline WFIRS-P score, mean (SD)	
	Responder	Non-responder	Responder	Non-responder
Family	16.1 (6.05)	12.1 (7.75)	18.4 (6.60)	10.2 (6.78)
Learning and School	14.8 (6.27)	10.8 (5.62)	15.6 (5.55)	11.2 (5.85)
Life skills	13.9 (4.47)	9.6 (4.72)	14.4 (4.62)	9.9 (5.02)
Child’s self-concept	4.7 (1.77)	2.5 (2.30)	4.5 (1.84)	2.0 (1.99)
Social activities	9.1 (4.23)	6.3 (4.31)	11.3 (4.65)	5.7 (5.21)
Risky activities	8.0 (3.78)	4.1 (3.35)	7.5 (3.26)	3.4 (2.94)

For both studies, baseline differences in WFIRS-P scores between responders and non-responders were significant for all domains (all *p* < 0.001)

*SD* standard deviation, *WFIRS*-*P* Weiss Functional Impairment Rating Scale-Parent Report

### Stratification of baseline WFIRS-P scores by patient characteristic subgroups

#### Participants

The pooled data set from the seven studies comprised 2099 participants, with a mean age of 11.0 years (SD, 2.92). Of these, 563 (26.8%) were girls, 863 (41.1%) were enrolled in Europe and 359 (17.1%) had comorbid ODD. In total, 1803 (85.9%) participants were diagnosed with combined or predominantly hyperactive-impulsive type ADHD and 295 (14.1%) with predominantly inattentive type ADHD (information was not available for one individual from SPD489-325).

#### Effect of age

Younger age correlated weakly but significantly with worse symptoms and functioning in the Family and Social Activities domains, with low values of Pearson’s *r* (– 0.1104 and – 0.1483, respectively) (Table 4). A similar association was seen with ADHD-RS-IV and CGI-S scores (– 0.2809 and – 0.2137, respectively).

**Table 4:** Pearson correlation coefficients for mean baseline WIFRS-P and CGI-S scores and mean baseline ADHD-RS-IV total score with age

WFIRS-P domain	Pearson’s *r*
Family	− 0.1104*
Learning and School	− 0.0501
Life Skills	− 0.0458
Child’s Self-Concept	+ 0.0989*
Social Activities	− 0.1483*
Risky Activities	− 0.0607
CGI-S	− 0.2137*
ADHD-RS-IV	− 0.2809*

*ADHD*-*RS*-*IV* Attention-Deficit/Hyperactivity Disorder Rating Scale IV, *CGI*-*S* Clinical Global Impression-Severity, *WFIRS*-*P* Weiss Functional Impairment Rating Scale-Parent Report

*p < 0.001 (*p* values > 0.001 not shown)

#### Effect of sex

**Fig. 3:**
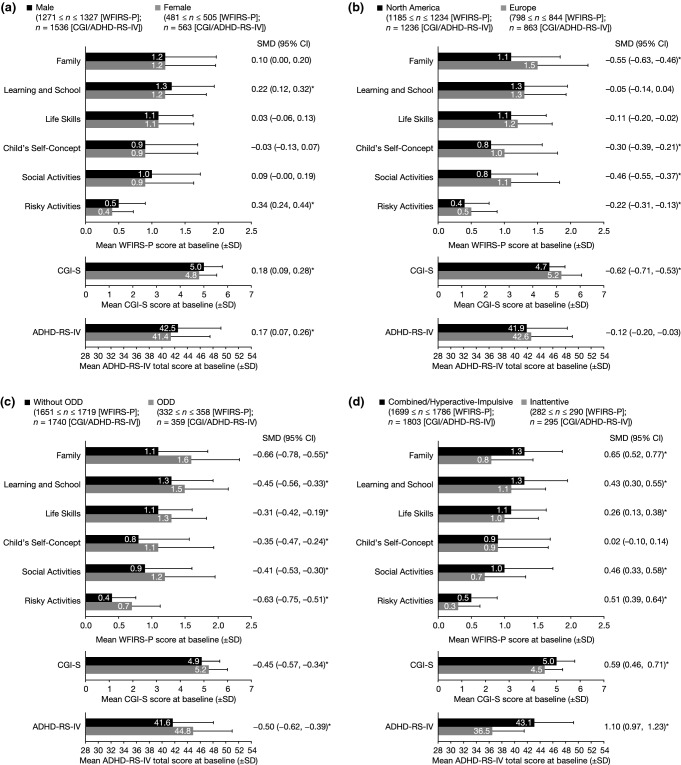
Baseline WFIRS-P scores stratified by **a** sex, **b** continent of enrolment, **c** ODD diagnosis and **d** ADHD presentation. *p < 0.001 (*p* values > 0.001 not shown). *ADHD* attention-deficit/hyperactivity disorder, *ADHD*-*RS*-*IV* Attention-Deficit/Hyperactivity Disorder Rating Scale IV, *CGI*-*S* Clinical Global Impression-Severity, *CI* confidence interval, *ODD* oppositional defiant disorder, *SD* standard deviation, *SMD* standardized mean difference, *WFIRS*-*P* Weiss Functional Impairment Rating Scale-Parent Report

Baseline WFIRS-P scores in most domains were similar amongst male and female participants (Fig. 3a). Scores were slightly worse in boys than in girls in the Risky Activities domain and Learning and School domain, with small SMDs (0.34 and 0.22, respectively). CGI-S scores and ADHD-RS-IV total scores were also slightly worse in boys than in girls, with small SMDs (0.18 and 0.17, respectively).

#### Effect of continent of enrolment

Baseline WFIRS-P scores were significantly worse in participants enrolled in Europe than in North America in all domains except for the Learning and School domain, and the Life Skills domain (Fig. 3b). SMDs between subgroups were largest in the Family and Social Activities domains (0.55 and 0.46, respectively). Baseline CGI-S scores were also significantly worse in participants enrolled in Europe than in North America, but ADHD-RS-IV total scores were similar. The SMD between subgroups was 0.62 for CGI-S score and 0.12 for ADHD-RS-IV total score.

#### Effect of ODD

Baseline WFIRS-P scores in all domains were significantly higher (worse) in participants with ODD and ADHD than in those without ODD (Fig. 3c). SMDs between the subgroups with ODD and without ODD subgroups were largest in the Family and Risky Activities domains (0.66 and 0.63, respectively). Baseline CGI-S scores and ADHD-RS-IV total scores were also significantly worse in participants with ODD and ADHD than in those without ODD. SMDs between subgroups were similar for CGI-S score and ADHD-RS-IV total score at baseline (0.45 and 0.50, respectively).

#### Effect of ADHD presentation

Baseline WFIRS-P scores were significantly worse in participants with combined or predominantly hyperactive-impulsive type ADHD than in those with predominantly inattentive type ADHD in all domains except for Child’s Self-Concept (Fig. 3d). SMDs between the subgroups were largest in the Family and Risky Activities domains (0.65 and 0.51, respectively). Baseline CGI-S scores and ADHD-RS-IV total scores were also significantly worse in participants with combined or predominantly hyperactive-impulsive type ADHD than in those with predominantly inattentive type ADHD. The SMD between subgroups was 1.10 for ADHD-RS-IV total score and 0.59 for CGI-S at baseline.

## Discussion

These post hoc analyses revealed that previously reported group-level improvements in WFIRS-P functional impairment did in fact translate into clinically relevant improvements in individual participants. Many participants receiving ADHD medications in two phase 3 clinical trials experienced meaningful and perceptible improvements in functioning, as measured using the WFIRS-P [11, 18]. These results support the recommendation that assessment and management of ADHD should include evaluation of each patient’s functional impairment using a reliable and responsive rating scale [15]. To this end, the MID provides a valuable tool for interpreting WFIRS-P response.

By stratifying baseline WFIRS-P scores by patient characteristic subgroups in seven phase 3 clinical trials, the present analyses also showed that children and adolescents with comorbid ODD tend to have worse functional impairment than those without comorbid ODD. Having combined type or hyperactive-impulsive type ADHD, being enrolled into a study in Europe, being male and being younger also had modest negative effects on baseline WFIRS-P scores.

In the responder analyses, the WFIRS-P Learning and School domain saw the greatest proportions of participants with above-MID improvements following treatment with LDX or OROS-MPH in study SPD489-325, or with GXR or ATX in study SPD503-316. Treatment with LDX, OROS-MPH and GXR (but not ATX) also led to improvements in the Family domain. The stimulants LDX and OROS-MPH were associated with improvements in the Risky Activities domain, whereas the non-stimulants GXR and ATX were associated with improvements in the Social Activities domain. This apparent difference between stimulants and non-stimulants is consistent with previous findings and may arise from factors including differing modes of action, as well as baseline variations in disease severity and characteristics in the study populations [15]. Of note, the groups of patients who responded in both studies had a greater mean impairment at baseline than non-responders. This observation could result from the greater window for improvement in those with highest WFIRS-P scores at baseline. Alternatively, regression towards the mean cannot be ruled out.

In an ADHD symptom-based responder analysis of SPD489-325, the differences in the proportion of responding patients between active medication and placebo were larger than those seen for any WFIRS-P domain. In the present analyses, the proportion of WFIRS-P responders was largest in the Learning and School domain with 60.0% for LDX and 18.4% for placebo. At endpoint in the symptom-based responder analysis, the percentages of patients categorized as responders were 74.2% for LDX, 55.9% for OROS-MPH and 10.7% for placebo. Although in these analyses, symptom response was not based on a MID, but was defined as a reduction of at least 30% in ADHD-RS-IV total score from baseline and a CGI-I score of 1 or 2 at endpoint [21]. Consistent with the observation that placebo-adjusted response rates were generally higher for ADHD-RS-IV/CGI-I based criteria than WFIRS-P response rates, mean changes in WFIRS-P domain scores correlated moderately with mean changes in ADHD symptom-based scores in a previous post hoc analysis. An investigation of the associations between different symptom-based and non-symptom-based outcomes found that the ADHD-RS-IV and the WFIRS-P assess partially intersecting but distinct aspects of the response to pharmacological treatment [15]. The nature of functional impairment varies from patient to patient, which may explain why the observed effect sizes were lower for the WFIRS-P than the ADHD-RS-IV. By definition, all participants had poor symptom scores at baseline, but not all necessarily had impairment in every WFIRS-P domain. As such, the WFIRS-P may help to guide clinical management of individual patients by providing a way to identify specific domains of impairment.

A recent study looked at the relationship between symptomatic improvement and functional improvement as measured by the WFIRS-P in an open-label study of OROS-MPH in children and adolescents with ADHD [24]. Significant improvements in all WFIRS-P domains were observed from baseline to end of open-label treatment (3 months). However, a substantial number of those considered to be symptomatic responders failed to show improvement in functioning. Symptomatic response was defined as a reduction of at least 30% in ADHD-RS-IV total score from baseline, and functional response as a mean change from baseline in WFIRS-P score of 0.25 [24]. These results further highlight the lack of complete alignment between symptom improvement and functional improvement.

In the present analyses of baseline scores stratified by patient characteristic subgroups, differences between subgroups in WFIRS-P scores were generally similar in magnitude and direction to the differences in clinical symptom and severity scores. This relationship varied amongst WFIRS-P domains, however, with larger differences in the WFIRS-P Family and Risky Activities domains than in CGI-S score and ADHD-RS-IV total score differences between participants with and without ODD. In contrast, differences in the Life Skills, Child’s Self-Concept and Social Activities domains were smaller than for CGI-S and ADHD-RS-IV. These observed differences between WFIRS-P scores and ADHD-RS-IV and CGI-S scores further demonstrate the intersecting but distinct aspects of the response to treatment captured by the different instruments [15].

Of the factors tested, ODD diagnosis had the largest influence on pooled baseline WFIRS-P scores and was associated with worse scores across all six domains. ADHD-RS-IV and CGI-I baseline scores were also worse in participants with ODD than in those without ODD, which is consistent with ODD and ADHD having some similar and overlapping features. The results of the present study likely reflect greater severity of both ADHD symptoms and related functional impairment amongst participants with ADHD and ODD than in those without ODD.

ADHD subtype had a modest influence on functional impairment and individuals with combined/hyperactive-impulsive ADHD scored worse in most domains, particularly in the Family and Risky Activities domains, than those with predominantly inattentive ADHD. The effect of ADHD subtype was greatest on ADHD-RS-IV baseline score (as expected, given that individuals with combined type ADHD have a potentially broader range of symptoms than those with inattentive or hyperactive-impulsive type). The effects of age and sex on baseline WFIRS-P, ADHD-RS-IV and CGI-S scores were small. Respondents may not have scored impairments equally in patients of different ages. The requirement for CGI-S scores of four or more in many of the trials included in the pooled data set may have led to exclusion of girls with very mild functional impairment.

The worse baseline WFIRS-P scores observed in Europe than in North America could be caused by the pooling of several studies with different enrolment criteria; for example, the minimum baseline ADHD-RS-IV total score varied between 28 and 32, but could also reflect a higher threshold for diagnosis in Europe than in North America. A telephone survey conducted in the USA in 2011 revealed that 11% of US school-aged children had received a diagnosis of ADHD [25], whereas the pooled worldwide prevalence is estimated to be 5.29% [26]. However, a meta-regression analysis showed that the prevalence estimates for ADHD in Europe and North America were not significantly different [27]. Given the potential differences observed in the present study, it is perhaps a topic worthy of further investigation.

A key strength of the responder analyses was the use of published MID values to define WFIRS-P response. Information about individual response is important to clinicians, in addition to what is known about group-level responses to treatment. Analysis of raw scores may not be the best way to tell if an individual has improved. To this end, MIDs provide an important way to interpret patient-reported outcomes, which are often the outcomes of most importance to patients and their families [28]. A further strength is the use of MIDs derived from an anchor method, which indicate the perceived importance of a change, rather than distributional methods that are based solely on variation around the group mean [16]. These MIDs were based on estimates calculated in a naturalistic community sample with no intervention, thereby reducing potential bias [16]. Further strengths include the size of the datasets analysed, with the responder analyses including over 300 participants in each trial and the pooled baseline impairment analyses including over 2000 participants. In the responder analyses, the proportions of stimulant-naïve participants were similar across all treatment groups, suggesting that previous drug status is unlikely to affect the treatment groups differentially. Furthermore, response to LDX and GXR has been shown to be unaffected by prior stimulant treatment [18, 22].

The use of clinical trial populations was a key limitation of the present analysis. Pooling data from seven studies with different inclusion and exclusion criteria to increase statistical power may have confounded the analyses of baseline characteristics in subgroups. Notably, the response rates of active and placebo arms in SPD503-316 were higher than the response rates in SPD489-325, which is an argument against pooling outcome data. Another limitation of the present analyses is that clinical trial populations may not be representative of patients seen in general clinical practice. Further limitations include the short-term nature of the studies, which preclude evaluation of the long-term impact of ADHD treatments on functional outcomes. Improvements in functional impairment may develop over a longer period of treatment than relief of ADHD symptoms. Although using the parent-rated WFIRS-P overcomes possible unreliable self-rating by young people with ADHD, it also introduces the potential for misinterpretation of the affected individual’s true response. In addition, the studies described here were powered for the primary outcome of assessing efficacy using ADHD-RS-IV, whereas assessment of the WFIRS-P was a secondary outcome. Furthermore, studies SPD489-325 and SPD503-316 were not designed or powered for direct comparison of the test drugs (LDX and GXR, respectively) with the active controls (OROS-MPH and ATX, respectively). Finally, both the responder and baseline impairment analyses are limited by the risk of bias associated with post hoc analyses.

The effect of ADHD medications in different subgroups could not be assessed in the present analyses because the numbers of participants in subgroups within each study were too small for reliable comparisons. Previous subgroup analyses have assessed the efficacy of GXR, LDX and OROS-MPH in participants with and without previous exposure to stimulant ADHD medication [18, 22], as well as the efficacy of GXR in individuals with ADHD and oppositional symptoms [29]. Future studies could perhaps explore the effect of ADHD medications in different subgroups such as those described here. In addition, it would be interesting to explore the temporal profile of medication activity. Previous studies have shown GXR to result in consistent symptomatic responses, regardless of the time of administration [12, 29, 30]. Different formulations of psychostimulants, however, may differentially affect certain WFIRS-P domains depending on their duration of action. For example, if systemic concentrations of a medication have declined, then the potential benefits in the Family and Social Activities domains may be lost. Assessment of response in individuals receiving combination therapy would also provide insight into benefits provided by different mechanisms of action.

## Conclusion

In conclusion, the large placebo-adjusted effect sizes of ADHD medications in two short-term clinical studies did translate into clinically relevant improvements in WFIRS-P functional impairment scores for many patients receiving pharmacotherapy with LDX, OROS-MPH, GXR and ATX in the present responder analyses. Longer-term studies may be needed to reveal the treatment effects of ADHD medication on functional impairment beyond what has been shown here. Functional impairment is a diverse and subjective construct that is influenced by multiple factors including comorbidity, age, sex and geography. Not only relief of ADHD symptoms, but also a reduction of patients’ related but distinct functional impairments, is achievable with medication in many children and adolescents with ADHD. As such, optimal management should involve monitoring improvement in functioning and quality of life, as well as symptomatic improvement.
